# Temporally increasing light intensity produces similar lettuce growth more efficiently than fixed high light

**DOI:** 10.3389/fpls.2026.1763702

**Published:** 2026-02-04

**Authors:** Iro Kang, Sarah Ding, Qingwu Meng

**Affiliations:** Department of Plant and Soil Sciences, University of Delaware, Newark, DE, United States

**Keywords:** controlled environment agriculture, dynamic lighting, hydroponics, indoor vertical farming, LEDs, photobiology, PPFD

## Abstract

Sole-source light-emitting diode (LED) lighting is essential in growth chambers, indoor farms, and spaceflight settings. Although lettuce (*Lactuca sativa*) is typically grown under fixed photosynthetic photon flux densities (PPFDs), dimmable LEDs enable dynamic lighting strategies that may improve light use efficiency. However, plant responses to temporally varying PPFDs remain insufficiently characterized. An indoor experiment was conducted to determine how fixed PPFDs and temporal PPFD alternations influenced growth, morphology, and pigmentation of red-leaf lettuce ‘Rouxai’. From day 0 to 28, we grew lettuce hydroponically at 21–23 °C air temperature and 18%–27% relative humidity under six lighting treatments, including two fixed PPFDs of 150 and 350 µmol m^–2^ s^–1^ and four temporal PPFD alternations with increasing PPFDs (150→250→250, 150→350→350, 250→250→350, and 250→350→350 µmol m^–2^ s^–1^) over three phases [lag phase (days 0–11)→exponential phase (days 11–25)→finish phase (days 25–28)]. All treatments had the same light spectrum (50% warm white + 50% red) and 24-h photoperiod. Increasing the fixed PPFD from 150 to 350 µmol m^–2^ s^–1^ increased seedling shoot fresh and dry mass by 69% and 84%, respectively, leaf number from 4 to 5, leaf width by 22%, chlorophyll concentration index by 15%, and red coloration, while decreasing leaf length by 11%. Similarly, for mature plants, increasing the fixed PPFD from 150 to 350 µmol m^–2^ s^–1^ increased shoot fresh and dry mass by 66% and 70%, respectively, leaf number by 23%, leaf width by 11%, and chlorophyll concentration index by 37%, while decreasing light use efficiency by 27%–29%. Compared to the fixed 350 treatment, the 250→250→350 and 250→350→350 alternations resulted in similar biomass, morphology, and chlorophyll concentration. However, the 250→250→350 alternation had 23%–31% higher light use efficiency than the fixed 350 treatment. Increasing the total light integral from 363 to 847 mol m^–2^ increased shoot fresh and dry mass but decreased light use efficiency. In conclusion, a temporal light intensity alternation produces comparably high biomass in lettuce more efficiently than fixed high light.

## 1 Introduction

The global demand for fresh, nutritious, and locally produced vegetables has accelerated the expansion of controlled environment agriculture (CEA) systems such as indoor vertical farms and plant growth chambers (Ting et al., 2016). These systems provide precise control over environmental conditions including temperature, humidity, CO_2_ concentration, and light quantity, quality, and duration, which allows year-round production independent of external climatic fluctuations (Benke and Tomkins, 2017). Among these environmental conditions, light is a critical factor of plant growth and morphogenesis, and in fully enclosed systems where sunlight is absent, light-emitting diodes (LEDs) serve as the sole-source of photosynthetically active radiation (Johkan et al., 2010). Sole-source LED lighting enables high energy efficiency, wavelength specificity, and fine-tuned control of spectral composition and intensity (Kang et al., 2013). Consequently, LED-based lighting has become indispensable not only for terrestrial indoor farming but also for space crop production, where light delivery must be efficient, reliable, and adaptive to limited power budgets (Massa et al., 2016).

Light intensity, often expressed as the photosynthetic photon flux density (PPFD), fundamentally governs the rate of photosynthesis in plants (Jeong et al., 2012). Photosynthesis exhibits a non-linear response to increasing light intensity. As the PPFD rises from low to optimum levels, the photosynthetic rate increases almost linearly due to enhanced photon absorption and carbon fixation; however, beyond the light saturation point, the rate plateaus because of biochemical and physiological limitations in the photosynthetic apparatus. At excessively high PPFDs, photoinhibition may occur, damaging photosystem II and leading to decreased photosynthetic efficiency (Fujii et al., 2016). Therefore, determining the optimal PPFD that maximizes photosynthesis without incurring energy waste or plant stress is essential for achieving both high yield and energy use efficiency in CEA systems.

Beyond its role in photosynthesis, the PPFD strongly influences plant morphology, biomass accumulation, and pigment composition (Jishi, 2018). In leafy greens such as lettuce (*Lactuca sativa*), increasing the PPFD generally enhances shoot fresh and dry mass, leaf thickness, and compactness by stimulating carbon assimilation and leaf expansion (Kelly et al., 2020). However, plants grown under excessively high PPFDs may exhibit smaller, thicker leaves, shortened petioles, and increased dry matter content due to carbon partitioning toward structural tissues (Shao et al., 2020). Light intensity also affects secondary metabolites such as anthocyanins, which are responsible for red coloration in pigmented lettuce cultivars. A higher PPFD promotes anthocyanin biosynthesis by upregulating genes involved in the flavonoid pathway, resulting in deeper red hues (Ma et al., 2021). Therefore, light intensity is a critical determinant of not only biomass production but also the visual and nutritional quality of leafy greens produced in controlled environments.

Although constant light intensities are commonly used throughout a crop’s production cycle, plant light requirements vary among developmental stages. For instance, lettuce seedlings have a relatively low photosynthetic capacity and light demand during the early lag phase and are typically grown under moderate PPFDs of approximately 200 µmol m^–2^ s^–1^, whereas mature plants in the exponential or finish phase exhibit higher photosynthetic potential and carbon demand and can effectively utilize substantially higher light intensities, commonly reported in the range of 350–600 µmol m^–2^ s^–1^ (Yan et al., 2019a; Zhou et al., 2019). Consequently, using a fixed high PPFD from germination to harvest may lead to unnecessary energy consumption during early stages when plants cannot efficiently utilize the supplied photons. Conversely, providing too little light during later stages can restrict biomass accumulation and limit pigment formation. Adjusting light intensity or spectral composition dynamically according to each plant growth stage, termed “dynamic lighting” or “temporal light modulation”, has thus emerged as a promising strategy to enhance light use efficiency while maintaining or improving yield and quality (Mäkinen et al., 2025).

Recent studies have explored various dynamic lighting approaches, including diurnal light fluctuation, gradual intensity ramping, and light-on-demand strategies based on photosynthetic responses. Some research has shown that temporally increasing the PPFD during the growth cycle can sustain biomass production while reducing total energy input compared with fixed high-intensity lighting. For example, tomato (*Solanum lycopersicum*) plants grown under increasing PPFD schedules demonstrated comparable yields but greater energy use efficiency relative to constant high PPFD treatments (Zheng et al., 2023). Recent work demonstrated that applying temporally variable light intensity in lettuce increased shoot biomass and light use efficiency compared with constant lighting (Lin et al., 2026). However, these studies typically employed relatively simple lighting schemes, such as two discrete light intensity levels, and did not explicitly design PPFD gradients across multiple developmental stages. Moreover, most prior studies have focused on fruiting crops or investigated daily or hourly fluctuations rather than gradual PPFD alternations across distinct growth phases. In leafy greens, especially lettuce, the understanding of how temporally varying light intensity affects growth, morphology, and pigment development across the crop cycle remains limited. Moreover, few studies have quantified the trade-off between light use efficiency and yield under different temporal lighting patterns, which is crucial for practical implementation in energy-intensive and energy-limited environments such as indoor vertical farms and space habitats.

The objective of this study was to characterize how temporally alternating light intensity affected growth, morphology, pigmentation, and light use efficiency of hydroponically grown red-leaf lettuce ‘Rouxai’. Based on developmental changes in canopy size and photosynthetic capacity across growth stages, we postulated that 1) decreasing the PPFD and thus the daily light integral (DLI) during the lag phase would optimize early energy allocation, thereby decreasing final biomass but increasing light use efficiency; 2) increasing the PPFD and thus the DLI during the exponential phase or finish phase would capitalize on the expanding canopy’s increased light interception and photosynthetic capacity, thereby enhancing final biomass and chlorophyll index; and 3) temporally increasing the PPFD and thus the DLI would align photon delivery with the exponential plant growth pattern to benefit from both higher light use efficiency under a lower PPFD in early growth and higher biomass under a higher PPFD in late growth, thereby maintaining comparable growth at higher light use efficiency compared to a fixed high PPFD.

## 2 Materials and methods

### 2.1 Experimental design and setup

We performed an indoor experiment on hydroponically grown lettuce plants inside a growth room with adjustable light-emitting diode (LED) fixtures (Phytofy RL; OSRAM, Munich, Germany) that allowed precise regulation of both light intensity and spectrum. We conducted the experiment twice over time as a randomized complete block design with two blocks (replications) and six lighting treatments per block in the same ambient environment (21–23 °C air temperature and 18%–27% relative humidity). In each replication, we randomized the locations of the six lighting treatments using six individual LED fixtures on two vertical shelves, with three treatments per shelf. The LED fixtures were separated with opaque black cloth to minimize cross-treatment light contamination. Data were collected on ten seedlings and six mature plants as subsamples per treatment per replication.

### 2.2 Lighting treatments

**Figure 1:**
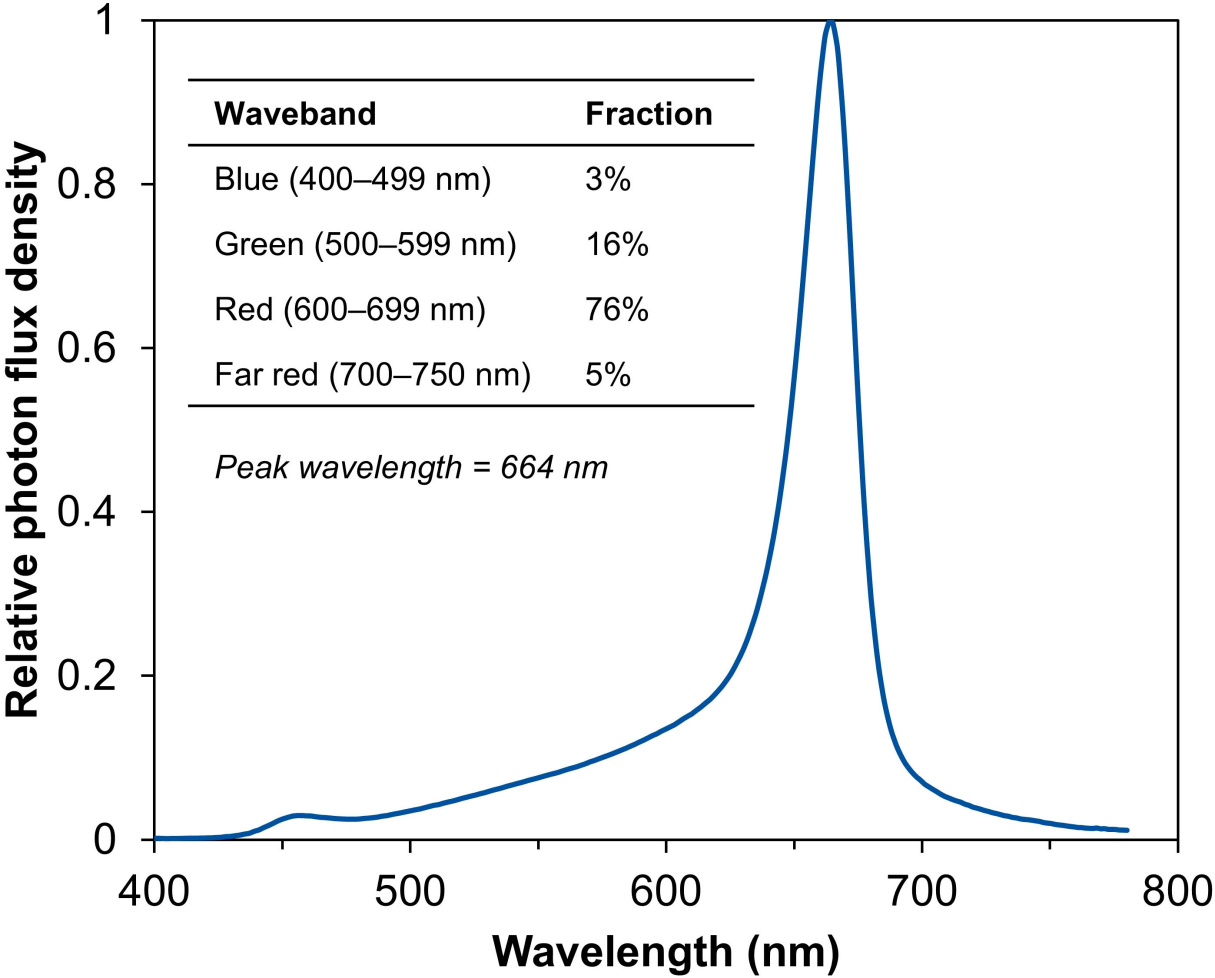
Spectral distribution of light-emitting diodes (50% warm white + 50% red) used in this study. Relative photon flux density is plotted against wavelength, with individual waveband fractions and the peak wavelength shown.

From seed (day 0) to final harvest (day 28), the six lighting treatments included two fixed PPFDs of 150 and 350 µmol m^–2^ s^–1^ and four temporal PPFD alternations with increasing PPFDs (150→250→250, 150→350→350, 250→250→350, and 250→350→350 µmol m^–2^ s^–1^) over three lettuce growth phases [lag phase (days 0–11)→exponential phase (days 11–25)→finish phase (days 25–28)] (**Table 1**). All treatments had the same light spectrum (50% warm white at 2700 K + 50% red) and 24-h photoperiod. We used a portable spectrometer (LI-180; LI-COR, Lincoln, NE, USA) and a quantum sensor (SQ-500; Apogee Instruments, Logan, UT, USA) to take light measurements at the plant level and achieved desired PPFDs using computer lighting software (Phytofy RL Software; OSRAM). When delivered alone, the warm-white LEDs emitted 7% blue light (400–499 nm), 29% green light (500–599 nm), 54% red light (600–699 nm), and 10% far-red light (700–750 nm). Based on spectroradiometric measurements, the resulting spectral distribution of 50% warm white + 50% red was approximately 3% blue light, 16% green light, 76% red light, and 5% far-red light, with a peak wavelength at 664 nm (**Figure 1**). We also placed two temperature and relative humidity sensors (HOBO MX1101 Temp/RH logger; Onset, Bourne, MA, USA) on the two shelves facing the aisle and enabled data logging at 1-h intervals.

**Table 1:** Six lighting treatments over three lettuce growth phases in 28 growing days under the same light spectrum (50% warm white at 2700 K + 50% red) and 24-h photoperiod.

Total light integral (mol m^–2^)	Photosynthetic photon flux density (µmol m^–2^ s^–1^)		
	Lag phase (days 0 to 11)	Exponential phase (days 11 to 25)	Finish phase (days 25 to 28)
Fixed			
363	150	150	150
847	350	350	350
Temporally alternating			
510	150	250	250
657	150	350	350
631	250	250	350
752	250	350	350

### 2.3 Plant material and seedling care

In each replication, on day 0, we sowed one seed of red-leaf lettuce ‘Rouxai’ (Johnny’s Selected Seeds, Winslow, ME, USA) in each of 180 2.54-cm-wide rockwool cubes (AO 25/40 Starter Plugs; Grodan, Milton, ON, Canada) pre-rinsed with tap water and moistened with a nutrient solution in six trays covered by transparent humidity domes (30 seeds per tray). Upon seed sow, the six trays were placed on top of six bus tubs under six lighting treatments. The humidity domes were removed on day 4. From day 4, we began to sub-irrigate seedlings as needed using a nutrient solution, which was made by dissolving a base fertilizer (12-4-16; JR Peters, Inc., Allentown, PA, USA) at a rate of 1.248 g L^–1^ and magnesium sulfate (JR Peters, Inc.) at a rate of 0.339 g L^–1^ sequentially in tap water. The nutrient composition in the solution contributed by the fertilizers was as follows (in mg L^–1^): 149 N, 22 P, 165 K, 87 Ca, 58 Mg, 47 S, 0.15 B, 0.56 Cu, 2.11 Fe, 0.62 Mn, 0.15 Mo, and 0.67 Zn. The nutrient solution pH was adjusted to ≈5.8 using diluted acid/base solutions (pH Down Phosphoric Acid and pH Up Potassium Hydroxide and Potassium Carbonate; General Hydroponics, Inc., Santa Rosa, CA, USA). The nutrient solution was replenished as needed using prepared nutrient solution of the same composition. Daily sub-irrigation ensured that the bottom quarter of the rockwool cubes was submerged.

### 2.4 Transplant and hydroponic production

On day 9 or 10, we sequentially dissolved the same two fertilizers at the same rates in each of the six bus tubs filled with tap water at a depth that submerged the bottom half of the six net cups inserted in each lid under each treatment. After the nutrient solutions were made, we adjusted pH to ≈5.8 using the same method as previously described. Each bus tub was randomly assigned to a lighting treatment. On day 11, data were collected from ten seedlings under each of the two fixed lighting treatments, and six seedlings per treatment were transplanted into each bus tub they were placed on top of. Seedling data collection was focused on the two fixed lighting treatments to isolate baseline light intensity effects prior to the initiation of temporal PPFD alternations; the four temporal PPFD treatments also had fixed PPFDs within the same range pre-transplant and only alternated PPFDs post-transplant. We inserted seedlings in rockwool into the net cups embedded in the lid on top of the bus tub. From day 11 to 28, we measured the pH, electrical conductivity (EC), and water temperature of all nutrient solutions daily, maintained the same pH range of 5.8 ± 0.3 as previously described, and replenished the nutrient solutions to the original depth with the same original nutrient solution as needed to keep roots submerged. As the nutrient solution was not fully replaced throughout the experiment, the nutrient concentration was intentionally not maintained with incremental nutrient solution top-offs to simulate real-world grower practices and conserve water and nutrients. The nutrient solution pH, EC, and temperature data from all treatments and replications are shown in **Table 2**. The nutrient solution temperature was 5–6 °C higher in Replication 1 than in Replication 2 due to the placement of bus tubs directly above heat-dissipating LED fixtures in Replication 1, but not in Replication 2. With water temperature as the blocking factor, lighting treatment responses were consistent across both replications. The air temperature and relative humidity (mean ± *SD*) on the two shelves ranged from 21.5 ± 0.4 °C to 22.4 ± 0.3 °C and from 18.1% ± 6.4% to 18.6% ± 6.7%, respectively, in Replication 1 and from 22.1 ± 0.3 °C to 22.5 ± 0.4 °C and from 26.2% ± 10.2% to 26.6% ± 10.4%, respectively, in Replication 2.

**Table 2:** pH, electrical conductivity (EC), and temperature of nutrient solutions throughout the experiment in Replications 1 and 2.

Photosynthetic photon flux density (µmol m^–2^ s^–1^)	pH	EC (dS m^–1^)	Temperature (°C)
Replication 1			
Fixed			
150→150→150	5.80 ± 0.08	2.38 ± 0.14	28.03 ± 0.38
350→350→350	5.81 ± 0.06	2.30 ± 0.13	29.18 ± 0.48
Dynamic			
150→250→250	5.84 ± 0.09	2.22 ± 0.18	28.72 ± 0.53
150→350→350	5.84 ± 0.09	2.02 ± 0.08	27.06 ± 0.39
250→250→350	5.82 ± 0.09	2.19 ± 0.13	28.01 ± 0.47
250→350→350	5.87 ± 0.08	2.08 ± 0.21	28.67 ± 0.99
Replication 2			
Fixed			
150→150→150	5.86 ± 0.10	1.88 ± 0.04	22.45 ± 0.34
350→350→350	5.84 ± 0.08	1.83 ± 0.08	23.20 ± 0.50
Dynamic			
150→250→250	5.83 ± 0.11	2.03 ± 0.02	22.67 ± 0.46
150→350→350	5.83 ± 0.09	1.95 ± 0.05	22.94 ± 0.55
250→250→350	5.86 ± 0.08	1.81 ± 0.13	23.06 ± 0.38
250→350→350	5.84 ± 0.09	2.00 ± 0.04	22.79 ± 0.57

### 2.5 Data collection and analysis

For seedlings sampled on day 11 (ten plants for each of two treatments), shoot fresh mass (g) was measured immediately after harvest. Shoot dry mass (g) was determined after drying the shoots at 60 °C for at least 5 d. The number of leaves was counted when individual leaves were longer than 0.5 cm. The length and width of the largest leaf (cm) were recorded using a ruler. Relative chlorophyll content was measured using a chlorophyll meter (MC-100; Apogee Instruments) by taking three readings (subsequently averaged for analysis) on the adaxial surface of fully expanded true leaves located in the upper canopy and not shaded by other leaves. Color indices (L*, a*, and b*) were determined from three readings (subsequently averaged for analysis) on randomly selected true leaf spots also fully exposed to light using a color reader (CR-10 Plus; Konica Minolta Sensing, Ramsey, NJ, USA).

For mature plants sampled on day 28 (six plants for each of the six treatments), shoot fresh mass (g) and shoot dry mass (g) were measured as described for seedlings. The number of leaves was counted when individual leaves were longer than 3 cm. The length and width of the largest leaf (cm) were measured with a ruler. Plant diameter (cm), defined as the longest horizontal distance between leaf edges, was recorded. Relative chlorophyll content was measured by taking three readings on randomly selected leaf spots fully exposed to light using the same chlorophyll meter. Light use efficiency was subsequently calculated as shoot fresh or dry mass divided by the total light integral (mol m^–2^) received over the entire experimental duration and normalized to the area occupied by each plant (0.0323 m^2^). Total light integrals were calculated by summing DLIs derived from pre-experiment PPFD measurements taken at the rockwool surface. These calculations did not account for changes in canopy-level PPFDs associated with increasing canopy height during plant growth. This approach is standard in sole-source LED studies with short-stature crops such as lettuce and allows consistent treatment comparisons, as reported previously (Kelly et al., 2020; Palmer and van Iersel, 2020). The area occupied by each plant was determined by dividing the total growing surface area of each bus tub directly irradiated by the LED fixture by the number of plants per bus tub following transplant.

**Figure 2:**
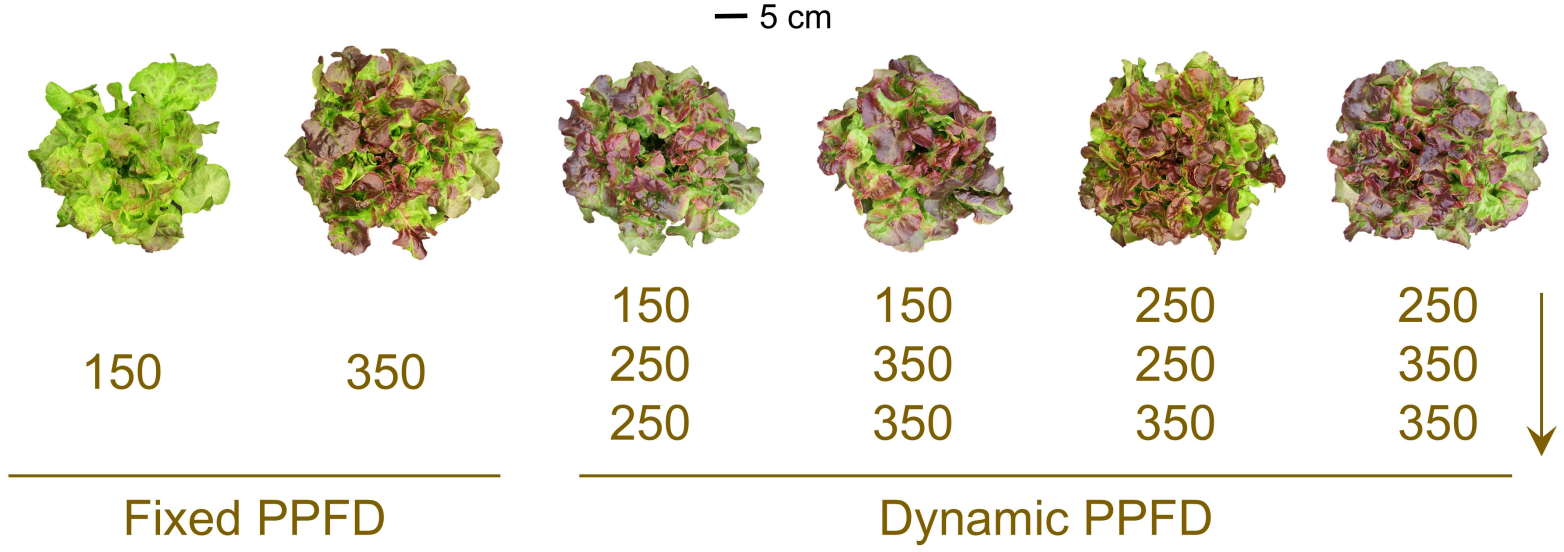
Representative red-leaf lettuce ‘Rouxai’ plants grown indoors hydroponically for 28 d after seed sowing from Replication 1. Plants received six lighting treatments, including two fixed photosynthetic photon flux densities (PPFDs) of 150 and 350 µmol m^–2^ s^–1^ and four temporal PPFD alternations with increasing PPFDs (150→250→250, 150→350→350, 250→250→350, and 250→350→350 µmol m^–2^ s^–1^) over three lettuce growth phases [lag phase (days 0–11)→exponential phase (days 11–25)→finish phase (days 25–28)]. All treatments had the same light spectrum (50% warm white + 50% red) and 24-h photoperiod.

We photographed a representative plant per treatment per replication to visually capture plant responses under the six lighting treatments, with photos from Replication 1 shown in **Figure 2**. We analyzed plant data in the statistical software JMP Pro (version 17.0.0; SAS Institute, Inc., Cary, NC, USA). Data were pooled from two replications for analysis given consistent treatment response treatments across replications and non-significant replication × treatment interactions. Pairwise comparisons were performed with Tukey’s honest significant difference test (*P* ≤ 0.05).

## 3 Results

### 3.1 Seedling growth responses

**Figure 3:**
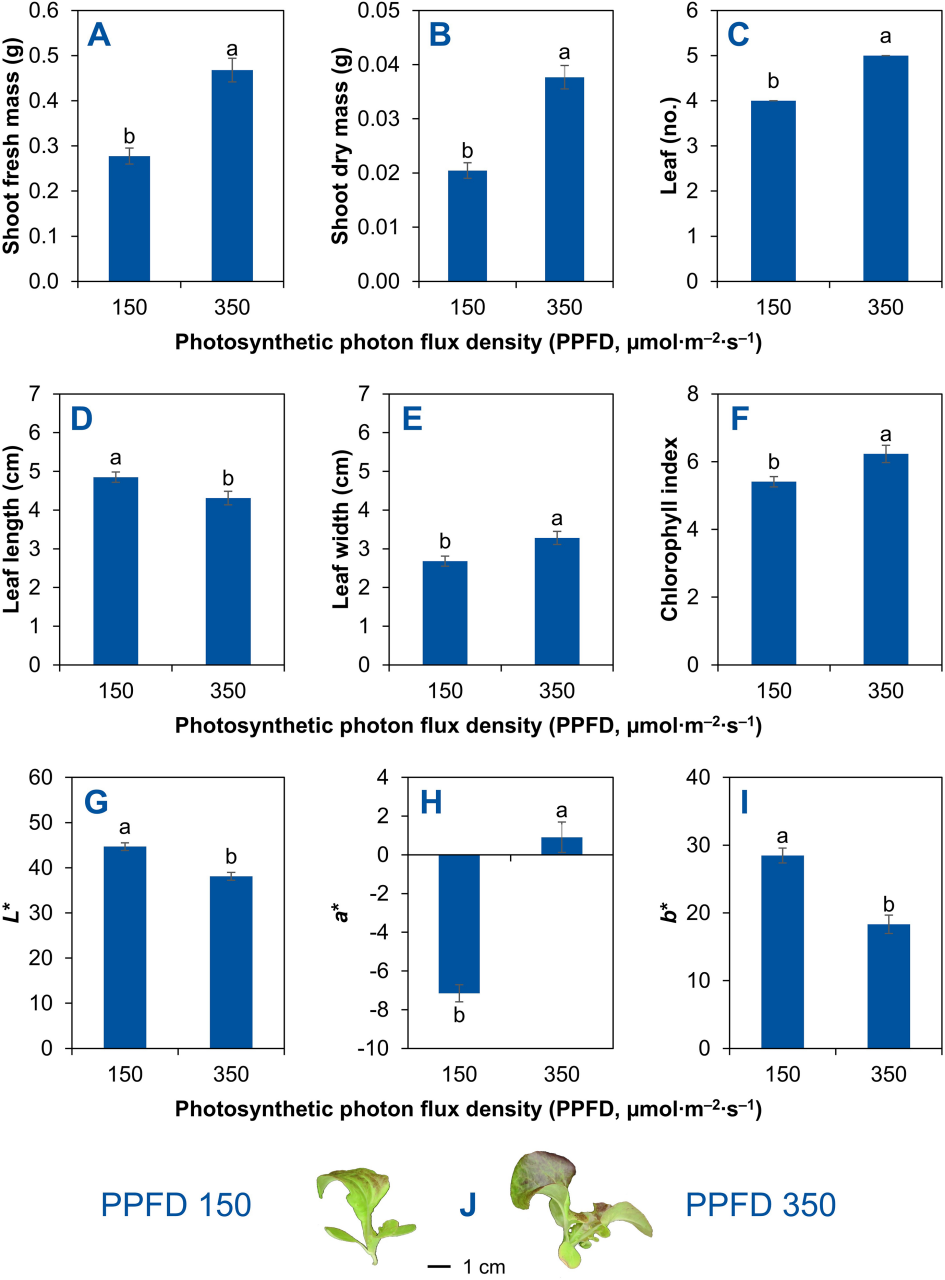
Shoot fresh mass **(A)**, shoot dry mass **(B)**, leaf number **(C)**, leaf length **(D)**, leaf width **(E)**, chlorophyll concentration index **(F)**, and leaf color indices, L* **(G)**, a* **(H)**, and b* **(I)**, of red-leaf lettuce ‘Rouxai’ seedlings grown indoors for 11 d after seed sowing under two fixed photosynthetic photon flux densities (PPFDs) of 150 and 350 µmol m^–2^ s^–1^. Both treatments had the same light spectrum (50% warm white + 50% red) and 24-h photoperiod. A representative seedling under each PPFD was photographed **(J)**. Means (± *SE*) within each parameter followed by different letters are statistically different based on Tukey’s honest significant difference test (*α* = 0.05). Data were pooled from two replications with 10 plant subsamples per treatment per replication.

Seedling biomass responded strongly to increasing PPFD. When the PPFD increased from 150 to 350 µmol m^–2^ s^–1^, shoot fresh mass was 69% greater (increased from 0.28 to 0.47 g; **Figure 3A**), and shoot dry mass increased by 84% (from 0.02 to 0.04 g; **Figure 3B**). Seedling morphology also shifted under the higher PPFD. Leaf number increased by 25% (from 4 to 5) as the PPFD increased from 150 to 350 µmol m^–2^ s^–1^ (**Figure 3C**). Additionally, leaf length decreased by 11% (from 4.9 to 4.3 cm; **Figure 3D**), whereas leaf width increased by 22% (from 2.7 to 3.3 cm; **Figure 3E**) as the PPFD increased from 150 to 350 µmol m^–2^ s^–1^.

For pigment-related parameters, the relative chlorophyll index was 15% higher (increased from 5.4 to 6.2) at 350 µmol m^–2^ s^–1^ compared with 150 µmol m^–2^ s^–1^ (**Figure 3F**). Regarding color indices, L* decreased by 15% (from 44.7 to 38.1) by increasing the PPFD from 150 to 350 µmol m^–2^ s^–1^, indicating darker foliage (**Figure 3G**). The a* value increased from –7.2 to 0.9 by increasing the PPFD from 150 to 350 µmol m^–2^ s^–1^, representing a change of approximately 113% relative to the absolute magnitude of the initial value (**Figure 3H**). The b* value decreased by 36% (from 28.5 to 18.3) as PPFD increased from 150 to 350 µmol m^–2^ s^–1^ (**Figure 3I**). **Figure 3J** presents photographs of representative seedlings from the 150 and 350 µmol m^–2^ s^–1^ PPFD treatments.

### 3.2 Mature plant growth responses

**Figure 4:**
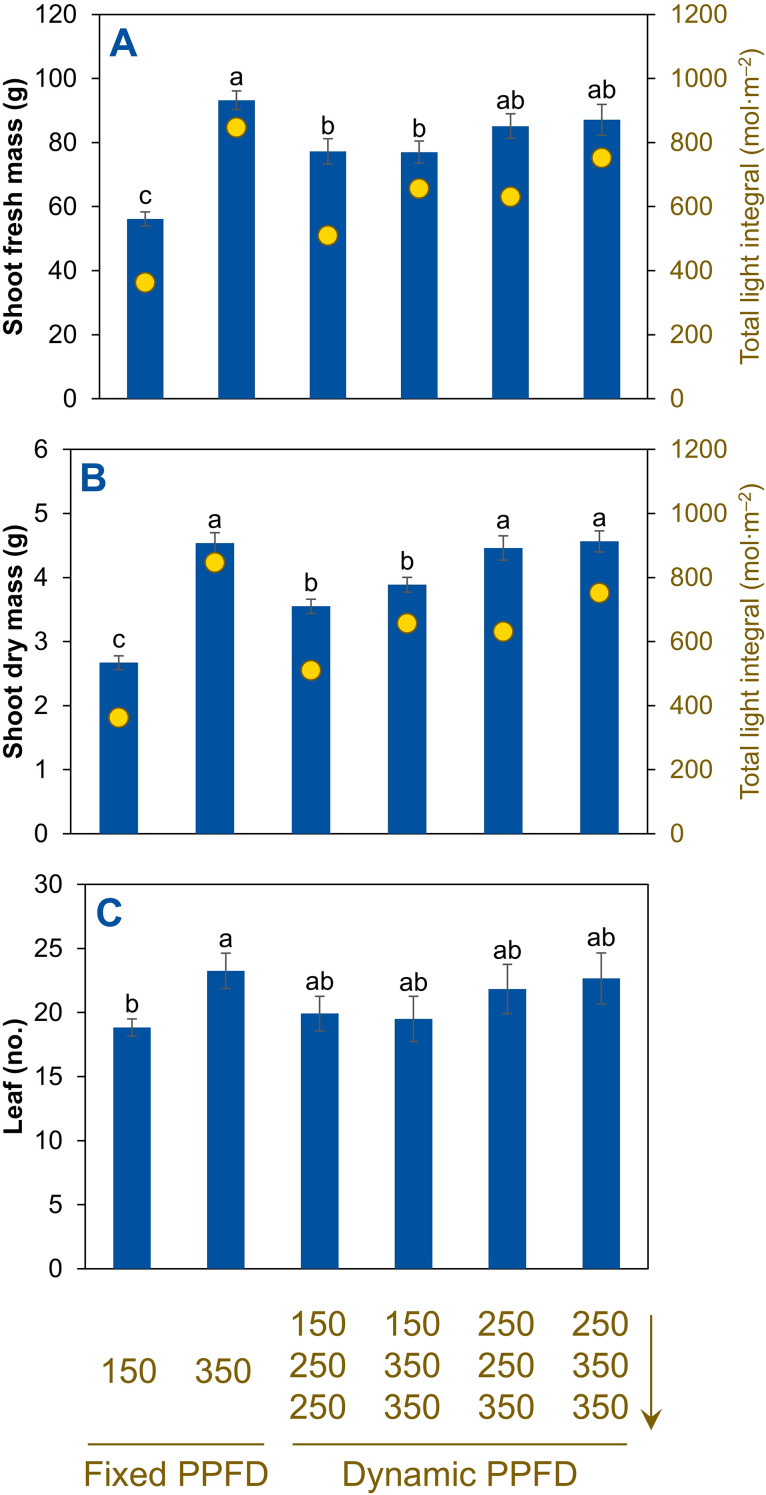
Shoot fresh mass **(A)**, shoot dry mass **(B)**, and leaf number **(C)** of red-leaf lettuce ‘Rouxai’ plants grown indoors hydroponically for 28 d after seed sowing. Plants received six lighting treatments, including two fixed photosynthetic photon flux densities (PPFDs) of 150 and 350 µmol m^–2^ s^–1^ and four temporal PPFD alternations with increasing PPFDs (150→250→250, 150→350→350, 250→250→350, and 250→350→350 µmol m^–2^ s^–1^) over three lettuce growth phases [lag phase (days 0–11)→exponential phase (days 11–25)→finish phase (days 25–28)]. All treatments had the same light spectrum (50% warm white + 50% red) and 24-h photoperiod. Means (± *SE*) within each parameter followed by different letters are statistically different based on Tukey’s honest significant difference test (*α* = 0.05). Data were pooled from two replications with six plant subsamples per treatment per replication. Total light integrals are shown on the secondary y axis as yellow dots overlaid in **(A)** and **(B)**.

Shoot fresh mass was lowest under the fixed PPFD of 150 µmol m^–2^ s^–1^ and was greater under all the other light treatments (**Figure 4A**). Shoot fresh mass increased by 66% (from 56.1 to 93.2 g) when the fixed PPFD increased from 150 to 350 µmol m^–2^ s^–1^. Similarly, shoot dry mass increased by 70% (from 2.7 to 4.5 g) as the fixed PPFD increased from 150 to 350 µmol m^–2^ s^–1^ (**Figure 4B**). The fixed PPFD at 350 µmol m^–2^ s^–1^ and the two temporal PPFD alternations at 250→250→350 and 250→350→350 µmol m^–2^ s^–1^ produced the greatest shoot dry mass, with no significant differences among these three treatments, whereas the fixed PPFD at 150 µmol m^–2^ s^–1^ produced the lowest.

**Figure 5:**
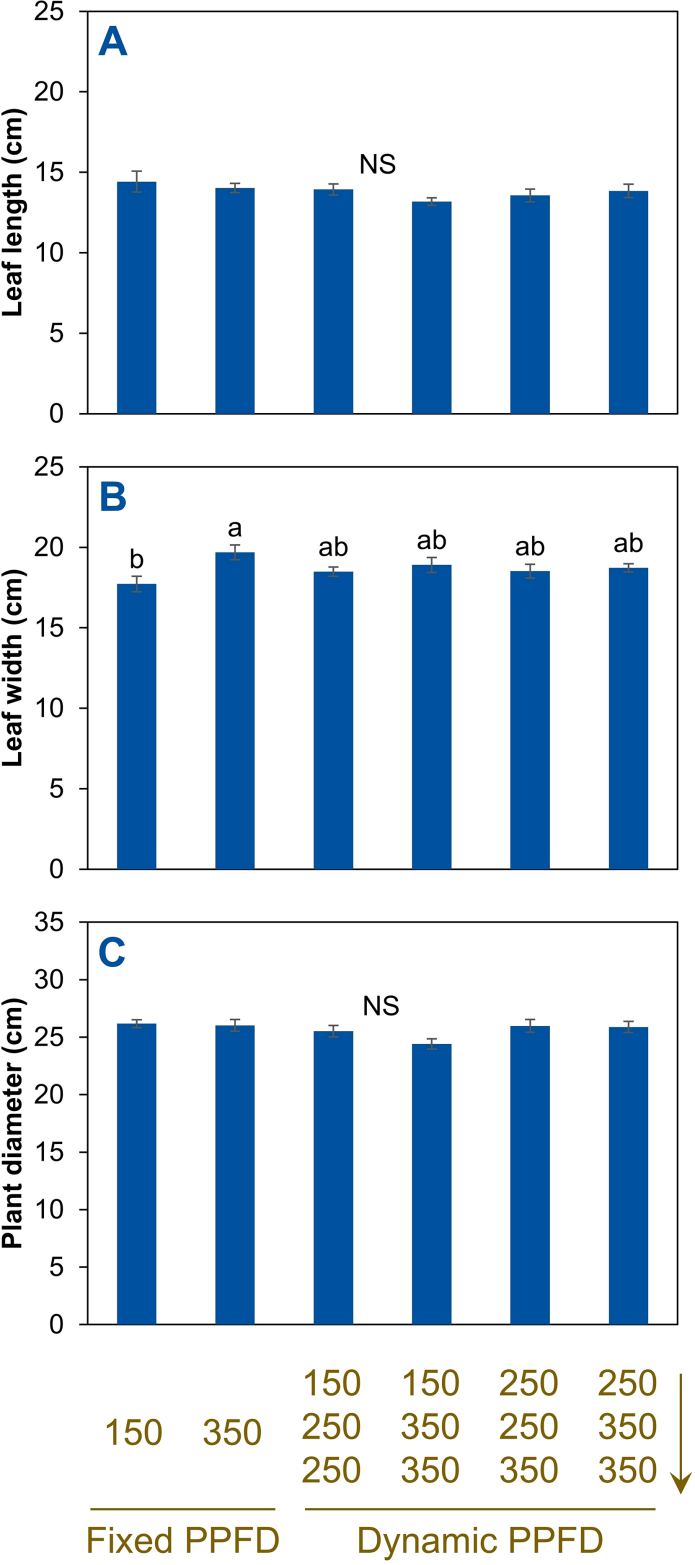
Leaf length **(A)**, leaf width **(B)**, and plant diameter **(C)** of red-leaf lettuce ‘Rouxai’ plants grown indoors hydroponically for 28 d after seed sowing. Plants received six lighting treatments, including two fixed photosynthetic photon flux densities (PPFDs) of 150 and 350 µmol m^–2^ s^–1^ and four temporal PPFD alternations with increasing PPFDs (150→250→250, 150→350→350, 250→250→350, and 250→350→350 µmol m^–2^ s^–1^) over three lettuce growth phases [lag phase (days 0–11)→exponential phase (days 11–25)→finish phase (days 25–28)]. All treatments had the same light spectrum (50% warm white + 50% red) and 24-h photoperiod. Means (± *SE*) within each parameter followed by different letters are statistically different based on Tukey’s honest significant difference test (*α* = 0.05). NS, non-significant. Data were pooled from two replications with six plant subsamples per treatment per replication.

Leaf number increased by 23% (from 19 to 23) as the fixed PPFD increased from 150 to 350 µmol m^–2^ s^–1^ (**Figure 4C**). All temporal (dynamic) PPFD alternating lighting treatments were intermediate and not statistically different from either. Leaf length showed no significant differences among treatments (**Figure 5A**). Leaf width increased by 11% (from 17.7 to 19.7 cm) by increasing the fixed PPFD from 150 to 350 µmol m^–2^ s^–1^ (**Figure 5B**). All temporal (dynamic) PPFD alternating lighting treatments were intermediate and statistically similar. There were no statistical differences among all six treatments for plant diameter (**Figure 5C**).

**Figure 6:**
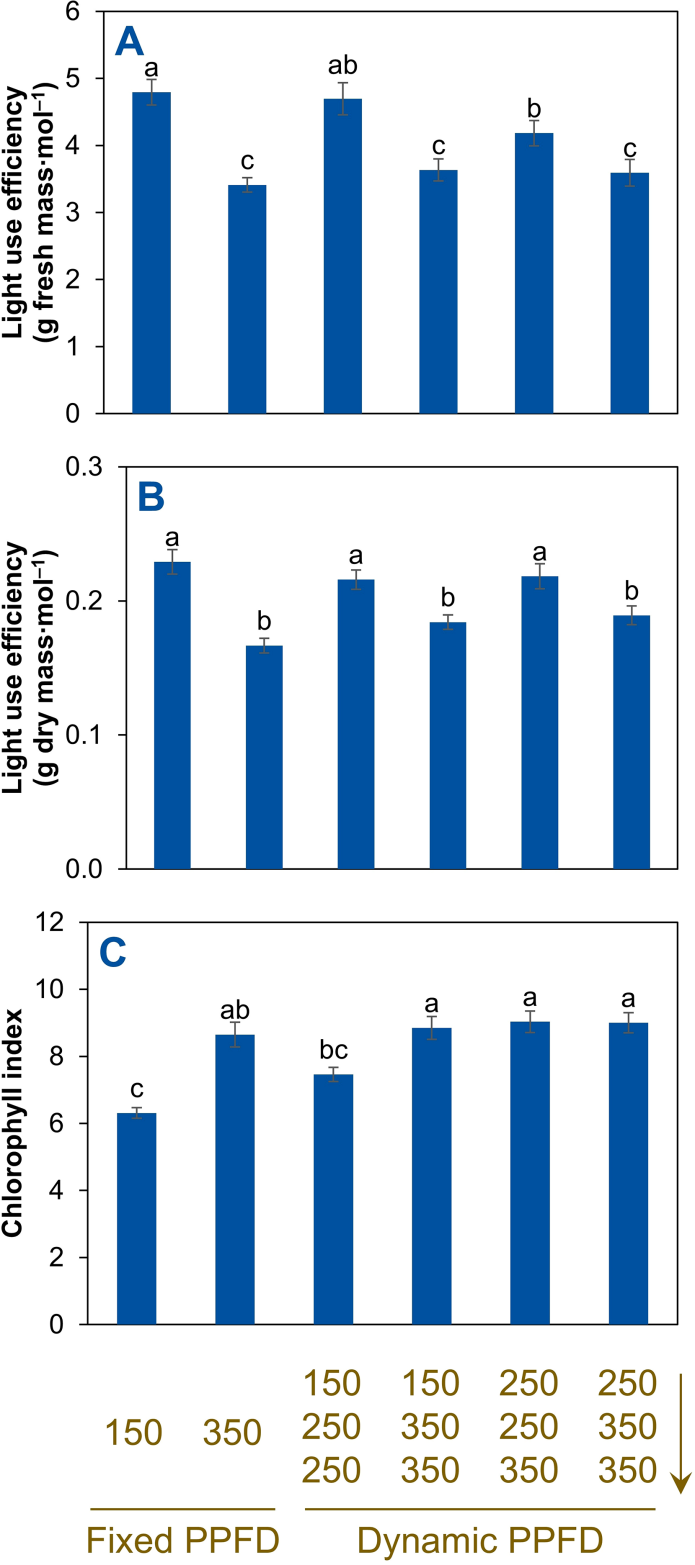
Light use efficiency based on shoot fresh mass **(A)** and shoot dry mass **(B)**, and chlorophyll concentration index **(C)** of red-leaf lettuce ‘Rouxai’ plants grown indoors hydroponically for 28 d after seed sowing. Plants received six lighting treatments, including two fixed photosynthetic photon flux densities (PPFDs) of 150 and 350 µmol m^–2^ s^–1^ and four temporal PPFD alternations with increasing PPFDs (150→250→250, 150→350→350, 250→250→350, and 250→350→350 µmol m^–2^ s^–1^) over three lettuce growth phases [lag phase (days 0–11)→exponential phase (days 11–25)→finish phase (days 25–28)]. All treatments had the same light spectrum (50% warm white + 50% red) and 24-h photoperiod. Means (± *SE*) within each parameter followed by different letters are statistically different based on Tukey’s honest significant difference test (*α* = 0.05). Data were pooled from two replications with six plant subsamples per treatment per replication.

Light use efficiency based on shoot fresh mass decreased by 29% (from 4.8 to 3.4 g fresh mass mol^–1^) by increasing the fixed PPFD from 150 to 350 µmol m^–2^ s^–1^ (**Figure 6A**). The highest light use efficiency (g fresh mass mol^–1^) occurred under the fixed PPFD at 150 µmol m^–2^ s^–1^, whereas the lowest occurred under the fixed PPFD at 350 µmol m^–2^ s^–1^ and the 150→350→350 and 250→350→350 µmol m^–2^ s^–1^ alternations. For light use efficiency based on dry mass (g dry mass mol^–1^), the fixed PPFD at 150 µmol m^–2^ s^–1^ and the 150→250→250 and 250→250→350 µmol m^–2^ s^–1^ alternations had higher efficiencies than the fixed PPFD at 350 µmol m^–2^ s^–1^ and the 150→350→350 and 250→350→350 µmol m^–2^ s^–1^ alternations (**Figure 6B**). Among the treatments that produced the highest shoot fresh and dry mass (350, 250→250→350, and 250→350→350 µmol m^–2^ s^–1^), the 250→250→350 µmol m^–2^ s^–1^ alternation exhibited significantly higher light use efficiency based on both fresh and dry mass compared to the 350 and 250→350→350 µmol m^–2^ s^–1^ treatments. The fixed PPFD at 150 µmol m^–2^ s^–1^ produced the lowest relative chlorophyll index (**Figure 6C**). The three temporal (dynamic) PPFD alternations of 150→350→350, 250→250→350, and 250→350→350 µmol m^–2^ s^–1^ had the highest relative chlorophyll index.

**Figure 7:**
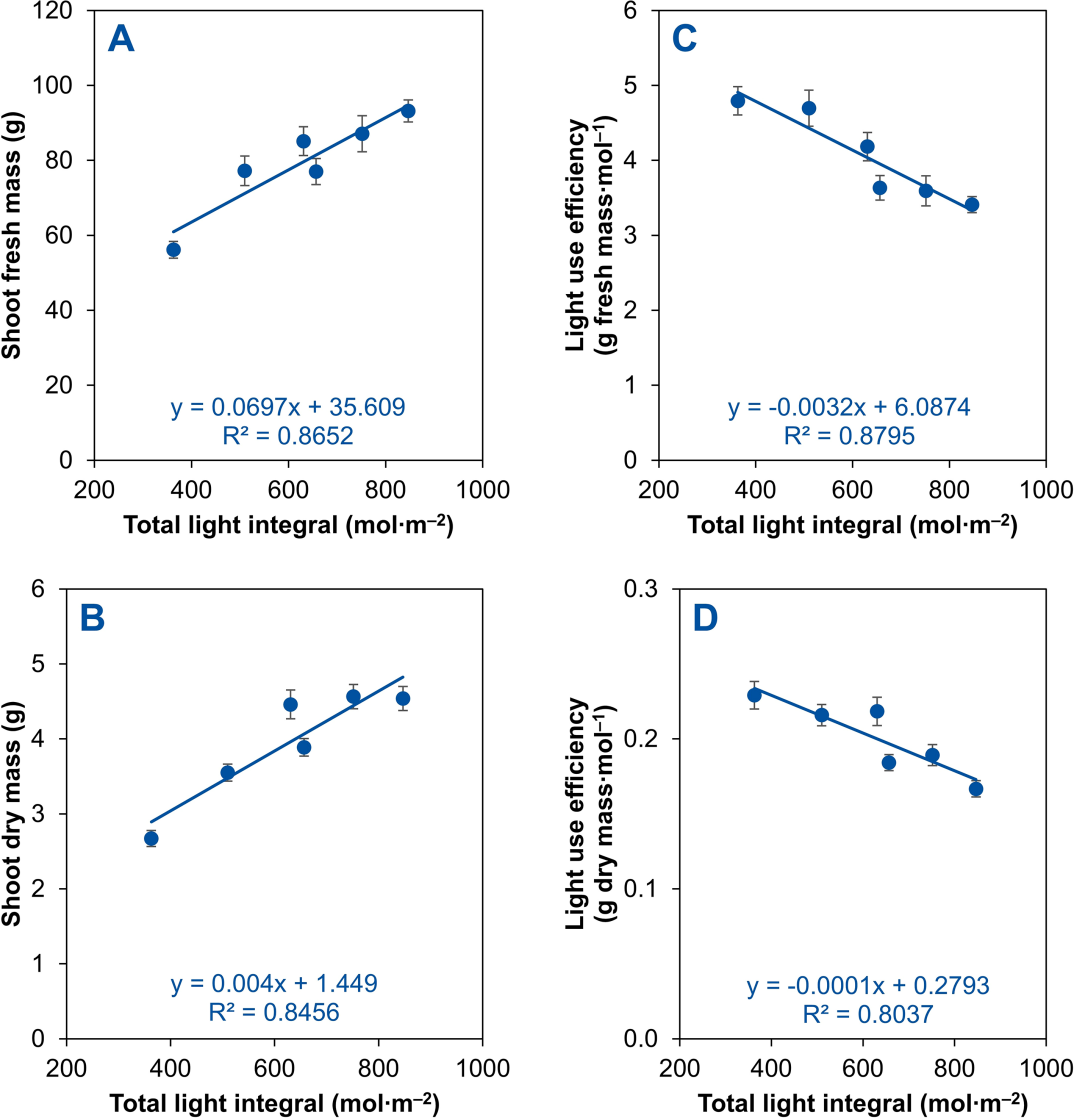
Linear relationships between the total light integral and shoot fresh mass **(A)**, shoot dry mass **(B)**, light use efficiency based on shoot fresh mass **(C)**, or light use efficiency based on shoot dry mass **(D)** of red-leaf lettuce ‘Rouxai’ plants grown indoors hydroponically for 28 d after seed sowing. Plants received six lighting treatments, including two fixed photosynthetic photon flux densities (PPFDs) of 150 and 350 µmol m^–2^ s^–1^ and four temporal PPFD alternations with increasing PPFDs (150→250→250, 150→350→350, 250→250→350, and 250→350→350 µmol m^–2^ s^–1^) over three lettuce growth phases [lag phase (days 0–11)→exponential phase (days 11–25)→finish phase (days 25–28)]. All treatments had the same light spectrum (50% warm white + 50% red) and 24-h photoperiod. Data were pooled from two replications with six plant subsamples per treatment per replication.

Across all fixed and temporal (dynamic) lighting treatments, shoot fresh and dry mass increased linearly as the total light integral increased from 363 (under the fixed PPFD at 150 µmol m^–2^ s^–1^) to 847 mol m^–2^ (under the fixed PPFD at 350 µmol m^–2^ s^–1^) (**Figures 7A, B**). Shoot fresh mass increased from 56.1 g at the lowest total light integral to 93.2 g at the highest, with a strong positive correlation (**Figure 7A**). Similarly, shoot dry mass increased from 2.7 g to 4.6 g as total light integral rose, with a positive correlation (**Figure 7B**). In contrast, light use efficiency for both shoot fresh and dry mass decreased linearly with increasing total light integrals, indicating diminishing biomass accumulation per unit of light received at higher total light integrals (**Figures 7C, D**). Light use efficiency for shoot fresh mass declined from 4.79 g mol^–1^ at the lowest total light integral to 3.41 g mol^–1^ at the highest total light integral (**Figure 7C**). Similarly, light use efficiency for shoot dry mass decreased from 0.23 to 0.17 g mol^–1^ as the total light integral increased from 363 to 847 mol m^–2^ (**Figure 7D**).

## 4 Discussion

### 4.1 Biomass and leaf morphological responses to increasing PPFDs

Across our six light treatments, shoot fresh and dry mass increased with the total light integral, while light use efficiency declined. Increasing the PPFD from 150 to 350 µmol m^–2^ s^–1^ in our experiment increased biomass of both seedlings and mature lettuce at 11 d and 28 d after seed sowing, respectively, consistent with prior reports that a higher DLI generally elevates lettuce yield and leaf expansion in controlled environments (Kelly et al., 2020). Also, the decline in light use efficiency for both shoot fresh and dry mass at higher total light integrals aligns with studies showing that light and electrical energy use efficiency decreased linearly with increasing DLI (Yan et al., 2019b).

Beyond biomass accumulation, light intensity differentially regulated leaf length and width, reflecting axis-specific sensitivities of cell division and expansion. In this study, while leaf length decreased in seedlings or remained stable in mature plants under higher light, leaf width generally increased. Similar trends have been observed in lettuce ‘Crunchy’, ‘Deangelia’, ‘Rex’, and ‘Rouxai’, where higher light intensity decreased or did not affect leaf length while increasing width (Kelly et al., 2020; Miao et al., 2023). This decoupling suggests that longitudinal and lateral growth are governed by distinct physiological mechanisms, a phenomenon known as growth polarity (Tsukaya, 2006). The increase in leaf width at higher PPFDs is likely driven by stimulated lateral cell division and expansion, supported by increased carbon availability (Milthorpe and Newton, 1963; Wilson, 1966). The reduction in leaf length at higher PPFDs indicates a suppression of the shade-avoidance response, where auxin-mediated cell wall loosening promotes longitudinal “stretching”, in favor of a “light-harvesting” strategy (Casal, 2012; Casal and Fankhauser, 2023). The stability of leaf length in mature ‘Rouxai’ suggests that longitudinal growth may be more strictly regulated by polar-specific genetic pathways (e.g., the ROTUNDIFOLIA pathway), whereas lateral expansion remains highly plastic to maximize the photosynthetic surface area (Tsukaya, 2006).

### 4.2 Dynamic PPFD strategies and canopy developmental alignment

The temporal (dynamic) lighting treatment of 250→250→350 µmol m^–2^ s^–1^ produced biomass comparable to the fixed PPFD at 350 µmol m^–2^ s^–1^ while improving light use efficiency, which is consistent with work showing that, at the same total light integral, gradually increasing the PPFD through the crop cycle enhances dry mass and thus light use efficiency compared to constant or decreasing light schedules (Jin et al., 2023). Mechanistically, increasing the PPFD later in the growth cycle coincides with larger canopies and greater light interception, which has been proposed as the basis for the advantage of gradually increasing light intensities in lettuce. Therefore, temporal (dynamic) or staged-intensity lighting approaches are viewed as practical methods to higher energy use efficiency without sacrificing yield in leafy greens. The lack of additional biomass accumulation when the PPFD was increased from 250 to 350 µmol m^–2^ s^–1^ during the exponential phase suggests that canopy photosynthetic capacity may have already approached saturation.

Gradually increasing the PPFD produces higher specific leaf area (SLA; leaf area/leaf mass) early in growth compared to a fixed PPFD. This means thinner leaves and more surface area per unit biomass, which increases light interception efficiency when canopy coverage is low. Later in the crop cycle, SLA decreases as the PPFD increases while differences among fixed high light and alternating lighting treatments diminish. At this stage, light interception is already high in all treatments, so early-stage differences in SLA are the main contributors to treatment differences in light use efficiency (Carotti et al., 2021).

An important mechanistic explanation for the improved performance of temporally increasing PPFD strategies lies in their alignment with photosynthetic capacity and light use efficiency under varying irradiance. The observed negative linear relationship between the total light integral and light use efficiency reflects diminishing biomass returns as the instantaneous PPFD increasingly exceeds canopy photosynthetic capacity. Under high irradiance, photosystem II becomes saturated and quantum efficiency declines, causing a greater proportion of absorbed photons to be dissipated through non-photochemical quenching (NPQ) rather than used for carbon fixation (Kaiser et al., 2018). These photosystem limitations reduce biomass gain per unit of delivered light, explaining the decline in light use efficiency at higher total light integrals. Conversely, distributing the same DLI across a longer photoperiod at a lower instantaneous PPFD has been shown to reduce NPQ, maintain photosystem II closer to its optimal operating range, and enhance daily electron transport rate, thereby improving biomass accumulation per unit of delivered light (Elkins and van Iersel, 2020; Palmer and van Iersel, 2020). Meng and Severin (2024) tested baby greens under the same DLI (17.28 mol m^–2^ d^–1^) with diurnal (12-h day/12-h night; 400/0 µmol m^–2^ s^–1^), alternating (12-h day/12-h day; 300/100 µmol m^–2^ s^–1^), and continuous light (24-h day; 200 µmol m^–2^ s^–1^). By lowering peak instantaneous irradiance and extending the photoperiod, continuous light significantly increased light use efficiency in lettuce and kale (*Brassica oleracea* var. *acephala*), relative to diurnal light. This was attributed to improved photosynthetic efficiency as reduced NPQ and more stable operation of photosystem II allowed a greater proportion of absorbed photons to be used for carbon fixation rather than dissipated as heat or fluorescence.

In our experiment, temporal PPFD alternations such as 250→250→350 µmol m^–2^ s^–1^ appear to function comparable mechanisms. Moderate PPFDs during early growth minimized photon waste on small canopies and reduced unnecessary activation of NPQ. As the crop developed, the later increase to 350 µmol m^–2^ s^–1^ aligned with larger leaf area and greater chlorophyll pools capable of effectively processing additional photons. The resulting improvements in biomass per mol of light delivered are therefore attributable to higher photosynthetic efficiency during more of the crop cycle, consistent with a previous report that temporally increasing PPFD modulation increases energy use efficiency in leafy greens (Jin et al., 2023).

Although our study used a 24-h photoperiod to exclude dark-period effects and clearly examine how temporal PPFD changes alone influence lettuce growth and light use efficiency, the concept of dynamic light modulation can also be implemented under non-continuous lighting. Research has shown that increasing DLI through extended photoperiods or supplemental lighting improves lettuce biomass and yield in greenhouse systems with non-continuous light regimes (e.g., supplemental 0-, 4-, and 8-h lighting after sunset; Ayankojo et al., 2025). Furthermore, LED strategies that combine intensity and photoperiod can enhance light use efficiency in leafy greens by optimizing photon distribution over time, even when complete continuous lighting is not used (Boucher et al., 2023). These findings indicate that the advantages of dynamic PPFD strategies are not limited to 24-h lighting but are also applicable to commercial systems using non-continuous light regimes.

### 4.3 Pigmentation under temporal PPFD alternations

The L* value represents darkness and lightness, ranging from black (L* = 0) to white (L* = 100) (Owen and Lopez, 2015). The a* value is on a scale of greenness (negative) to redness (positive), whereas the b* value is on a scale of blueness (negative) to yellowness (positive). For pigmentation responses, the higher PPFD enhanced relative chlorophyll index and a* (intensified redness) in seedlings at 11 d after seed sowing. At 28 d after seed sowing, the higher fixed PPFD at 350 µmol m^–2^ s^–1^, and higher PPFD alternation at 150→350→350, 250→250→350, and 250→350→350 µmol m^–2^ s^–1^ increased relative chlorophyll index, similar to studies showing that higher PPFD increased chlorophyll content (Hwang et al., 2016; Park and Lee, 1999; Yeo et al., 2017). Higher light intensity increased nitrogen uptake in lettuce (Zhou et al., 2019), probably due to enhance chlorophyll content and electron transport capacity (Evans, 1989). This physiological response helps explain the observed increase in shoot fresh and dry mass under higher light intensity.

Although L*, a*, and b* color indices were not measured for mature plants in this study, the observed increase in a* (redness) in seedling under higher PPFD suggests enhanced anthocyanin accumulation during early growth. Anthocyanin synthesis is known to be stimulated by high light intensity through the upregulation of genes involved in the flavonoid biosynthesis pathway (Ma et al., 2021). Future studies can quantify color indices in mature plants to further elucidate the effects of dynamic lighting on pigmentation across developmental stages.

### 4.4 Experimental limitations

The 5–6 °C variation in nutrient solution temperature (28–29 °C in Replication 1 vs. 22–23 °C in Replication 2) highlights a common challenge in environmental control, which we addressed by treating replication as a blocking factor. In lettuce, root-zone temperatures near 24–25 °C are generally considered optimal when air temperatures are moderate (≈22 °C) (Thompson et al., 1998; Yamori et al., 2022). Since the nutrient solution temperature in Replication 1 was roughly 3–4 °C above this optimum and that in Replication 2 was 2–3 °C below it, the overall higher biomass and light use efficiency observed in Replication 1 suggest that the warmer root-zone temperature likely promoted root metabolic activity and nutrient uptake efficiency. While treating replication as a block accounts for these mean differences in growth magnitude, it may not fully capture potential non-linear interactions between root-zone temperature and PPFD.

Crucially, however, there was no evidence that the higher root-zone temperature exacerbated photoinhibition at high PPFDs. Photoinhibition typically manifests as a reduction in light use efficiency and carbon assimilation when high light energy exceeds the plant’s metabolic capacity (Müller et al., 2001), a process often worsened by heat stress (Takahashi and Murata, 2008). Had such an interaction occurred, the high-PPFD treatments in the warmer replication would have shown a relative decline in performance; instead, the higher absolute growth metrics in Replication 1 indicate that the plants maintained high photochemical efficiency. The fact that the directional trends for both plant biomass and morphology remained consistent between replications reinforces that the light intensity effects observed here are robust across a range of typical hydroponic thermal environments. Future studies should independently control root-zone temperature to better resolve specific light × temperature interactions.

### 4.5 Conclusions

Our findings demonstrate that maintaining a high PPFD throughout production was not necessary to achieve high yield and high-quality lettuce. Although the fixed 350 µmol m^–2^ s^–1^ treatment produced high biomass, two temporal PPFD strategies (250→250→350 and 250→350→350 µmol m^–2^ s^–1^) generated comparable biomass accumulation, morphological traits, and pigmentation. Among these, the 250→250→350 µmol m^–2^ s^–1^ alternation achieved similar biomass and quality while improving light use efficiency by 23%–31% relative to fixed high light at 350 µmol m^–2^ s^–1^. This outcome suggests that supplying a lower PPFD during early growth followed by a higher PPFD later in the cycle represents a rational lighting approach to enhancing crop productivity per unit of light delivered for leafy green production in controlled environment systems.

## Data Availability

The raw data supporting the conclusions of this article will be made available by the authors, without undue reservation.
